# The Features of Inflammation Factors Concentrations in Aqueous Humor of Polypoidal Choroidal Vasculopathy

**DOI:** 10.1371/journal.pone.0147346

**Published:** 2016-01-22

**Authors:** Jie Hu, Xuan Leng, Yijun Hu, Alp Atik, Xin Song, Zhixi Li, Yuhua Liu, Lin Lu

**Affiliations:** 1 State Key Laboratory of Ophthalmology, Zhongshan Ophthalmic Center, Sun Yat-sen University, Guangzhou, China; 2 Royal Victorian Eye and Ear Hospital, Melbourne, Australia; Massachusetts Eye & Ear Infirmary, Harvard Medical School, UNITED STATES

## Abstract

**Purpose:**

To investigate the cytokine concentrations in the aqueous humor of patients with refractory polypoidal choroidal vasculopathy (PCV).

**Methods:**

Three separate groups of patients were studied–refractory PCV (Group A, 41 eyes), stable PCV (Group B, 39 eyes) and senile cataract (Group C, 44 eyes). Aqueous humor samples were collected at two time points for Groups A and B–before the first intravitreal ranibizumab injection and before the last injection. Aqueous humor samples were collected prior to phacoemulsification in Group C. The cytokine concentrations of interleukin 2, 6, and 8 (IL-2, IL-6, and IL-8), tumor necrosis factor α (TNF-α), monocyte chemotactic protein 1 (MCP-1), and vascular endothelial growth factor (VEGF) were measured by cytometric bead array and flow cytometry.

**Results:**

Before the first treatment, the MCP-1, VEGF, and TNF-α levels in Group A were significantly higher than those in Group C (*P* < 0.05), and the MCP-1 and VEGF levels in Group A were significantly higher than those in Group B (*P* < 0.05). Significantly higher MCP-1 and VEGF levels were seen in Group B compared to Group C (*P* < 0.05). Before the final treatment, the MCP-1, VEGF, and TNF-α concentrations in Group A were significantly higher than those in Group B (*P* < 0.05) and Group C (*P* < 0.05). IL-2 levels were significantly lower in Group A compared to Group B (*P* < 0.05) and Group C (*P* < 0.05).

**Conclusion:**

Inflammatory cytokines such as MCP-1, VEGF, and TNF-α may be associated with the pathogenesis of both stable and refractory PCV.

## Introduction

Polypoidal choroidal vasculopathy (PCV) is a choroidal vascular disease characterized by a branching vascular network (BVN) and clinically visible orange–red subretinal nodules which originate from the choroidal vasculature [1]. PCV is often complicated with serous retinal detachment, retinal edema or hemorrhagic pigment epithelial detachment (PED). The prevalence of PCV is higher in Asians and Blacks than Caucasians [2, 3]. Although vertepofin photodynamic therapy (PDT), intravitreal ranibizumab (IVR) or a combination of both have been proposed for the treatment of PCV, recurrence has been noted in some patients [2–4]. The pathogenesis of PCV is still unclear, with a potential role for inflammation driven by both genetic and environmental factors [2, 3]. Different inflammatory biomarkers have been shown to be associated with PCV such as IL-23 and C-reactive protein (CRP) [5–8]. Moreover, some of these biomarkers have been associated with disease activity [5]. In the present study we showed elevated aqueous humor levels of tumor necrosis factor α (TNF-α), monocyte chemotactic protein 1 (MCP-1), and vascular endothelial growth factor (VEGF) in patients with both stable and refractory PCV compared to controls.

## Patients and Methods

### Patients

This retrospective study was guided by the tenets of the Declaration of Helsinki and approved by the ethics committee of the Zhongshan Ophthalmic Center of Sun Yat-Sen University, China. All subjects were Chinese and written informed consent was obtained from each patient after a detailed explanation of the purposes and risks of this study. PCV patients were recruited from the Zhongshan Ophthalmic Center between June 2012 and January 2015. Each patient underwent a complete ophthalmic examination, indocyanine green angiography (ICGA), fluorescein angiography (FA) and optical coherence tomography (OCT). The inclusion criteria were: 1) treatment-naive PCV, 2) presence of at least one orange subretinal nodule associated with submacular exudates and/or hemorrhage; 3) polyps and BVN detected by ICGA, 4) leakage on FA, subretinal fluid and/or hemorrhage on OCT. The exclusion criteria included: 1) diagnosis of other chorioretinal diseases such as wet age-related macular degeneration (wAMD), diabetic retinopathy and pathological myopia; 2) previous treatment for PCV; 3) hypertension, diabetes mellitus, autoimmune disease, or other systemic diseases; 4) previous intraocular surgery; 5) medications that may affect cytokine concentrations (e.g. steroids). After enrolment, each patient underwent initial treatment (IVR monotherapy or combination of PDT and IVR) and pro re nata (PRN) re-treatment (IVR monotherapy or the combination of PDT and IVR) based on clinical manifestations according to the EVEREST study and the guidelines suggested by the expert PCV panel [4, 9]. The follow-up interval for each patient was one month.

Refractory PCV patients were defined as those showing signs of recurrence or persistence of the lesions that met the re-treatment criteria of the EVEREST study and the expert PCV panel guidelines at 12 months follow-up after the latest treatment/re-treatment: 1) polyps that partially regressed or persisted on ICGA, 2) polyps that completely regressed on ICGA, but still leaked on FA with clinical or OCT signs of activity. Stable PCV patients were defined as those whose polyps completely regressed with no leakage on FA and no subretinal fluid on OCT at 12 months follow-up.

### Patient groups

Three separate groups of patients were studied–refractory PCV (Group A, 41 eyes), stable PCV (Group B, 39 eyes) and senile cataract (control) (Group C, 44 eyes). The senile cataract patients had no other ocular (wAMD, diabetic retinopathy, or pathological myopia) or systemic diseases (hypertension, diabetes mellitus, autoimmune disease).

### Aqueous humor acquisition

Aqueous humor samples (50–100 μl) were collected from patients with PCV via anterior chamber paracentesis with a 27G needle before IVR. Aqueous humor samples were collected before phacoemulsification in the senile cataract group. Samples were stored in sterilized 1.5ml Eppendorf tubes (Corning Inc., New York, NY, USA) at a temperature of –80°C.

### Measurement of multiple factors in the aqueous humor

Interleukin 2, 6, and 8 (IL-2, IL-6, and IL-8), MCP-1, TNF-α and VEGF in aqueous humor samples were captured by cytometric bead array (BD Bioscience, San Jose, CA, USA) according to the manufacturer's manual. Cytokine levels were then quantified by flow cytometry (BD FACSA; BD Bioscience, San Jose, CA, USA). Data analysis was performed with FCAP Array software (v3.0.1) (BD Bioscience, San Jose, CA).

### Statistical analysis

Data analysis of cytokine concentrations was performed with SPSS 16.0 (SPSS Inc., Chicago, IL, USA). Normality testing showed that cytokine levels did not bear normal distributions. Kruskal–Wallis H test was used to compare the differences in cytokine levels among the three groups. A *P-*value of < 0.05 was considered statistically significant in all tests. Bonferroni test was used to compare the cytokine levels between any two groups.

## Results

Demographic characteristics of the three groups of patients in the study are described in Table 1. Twenty-four patients in Group A and 19 patients in Group B received combined treatment of PDT and IVR (*P* = 0.501). The average number of PDT treatment for each patient (mean±SD) was 0.68±0.65 for Group A and 0.46±0.51 for Group B, respectively (*P* = 0.143). The average number of IVR treatment for each patient (mean±SD) was 3.42±0.48 for Group A and 1.17±0.09 for Group B, respectively (*P*<0.001). Seventeen patients in Group A and 20 patients in Group B received IVR monotherapy (*P* = 0.501). The average number of IVR treatment for each patient (mean±SD) was 4.00±0.12 for Group A and 2.14±0.10 for Group B, respectively (*P*<0.001).

**Table 1:** Demographic characteristics of patients.

Group	Age (yrs)	Gender	Treatment
	(Mean±SD)	(Males/Females)	IVR/PDT+IVR
A (41 eyes)	62.59±7.92	26 / 15	17 / 24
B (39 eyes)	62.56±9.67	21/ 18	20/ 19
C (44 eyes)	69.52±8.41	21/ 23	-/ -

**Fig 1:**
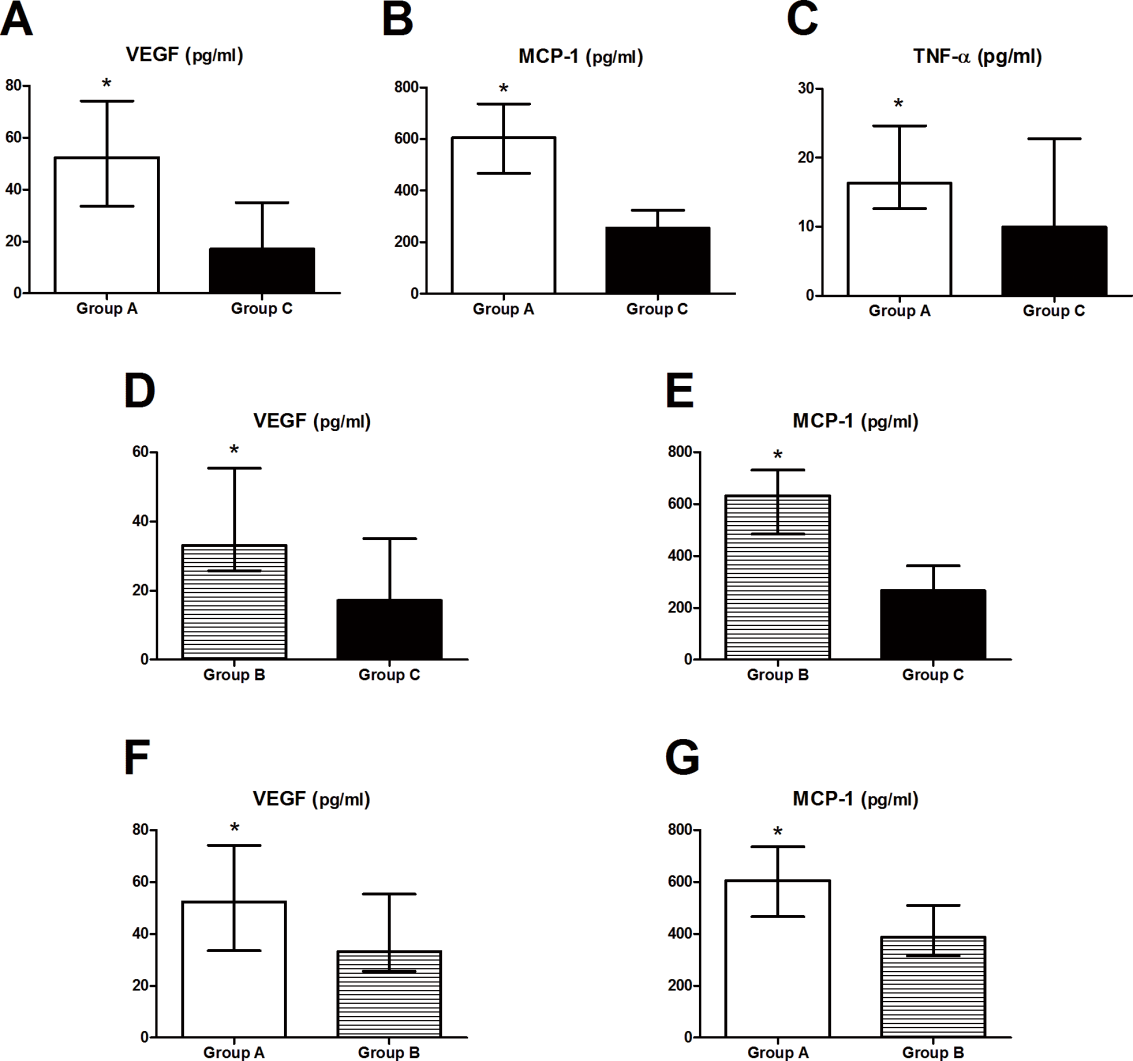
Aqueous humor concentrations of inflammatory cytokines prior to initial treatment in the three study groups (mean, median, standard deviation (SD)).

To investigate whether aqueous humor cytokines are markers of PCV, we compared the cytokine levels of the three groups. Aqueous humor samples for Group A (refractory PCV patients) and Group B (stable PCV patients) were collected prior to their initial treatment with IVR. Samples for Group C (senile cataract patients) were collected prior to phacoemulsification. Kruskal–Wallis H tests were performed to compare the six cytokine levels among the three study groups (Table 2). Our results showed that VEGF, MCP-1, and TNF-α levels in Group A were statistically higher than those in Group C (*P*_VEGF_ < 0.001, *P*_MCP-1_ < 0.001, and *P*_TNF-α_ = 0.011, respectively) (Fig 1A–1C). We also found that Group B had significantly higher VEGF and MCP-1 levels compared to Group C (*P*_VEGF_ < 0.001 and *P*_MCP-1_ < 0.001, respectively) (Fig 1D and 1E). VEGF and MCP-1 levels were significantly higher in Group A compared to Group B (*P*_VEGF_ = 0.027 and *P*_MCP-1_ < 0.001, respectively) (Fig 1F and 1G).

**Table 2:** Levels of inflammatory cytokines prior to initial treatment of PCV.

Group	(pg/ml)	IL-2	IL-6	IL-8	MCP-1	VEGF	TNF-α
Control	Mean	22.01	10.02	14.91	276.59	20.48	16.30
	Median	15.03	8.05	13.87	255.50	17.13	9.96
	SD	24.61	8.93	14.61	94.34	18.42	21.77
Pretreatment							
Group A	Mean	15.58	8.59	15.20	615.72	54.53	19.64
	Median	14.00	4.24	13.30	605.50	52.33	16.30
	SD	10.43	12.94	10.43	179.12	27.29	12.78
Group B	Mean	15.95	5.74	11.97	427.42	42.72	16.20
	Median	15.30	3.20	12.32	390.20	33.14	13.00
	SD	10.33	7.77	10.11	148.46	22.73	13.67
P value		0.580	0.071	0.331	<0.001*	<0.001*	0.029*

*P-*values correspond to Kruskal-Wallis H testing among the three groups.

*Statistically significant results in the three groups (*P* < 0.05).

**Fig 2:**
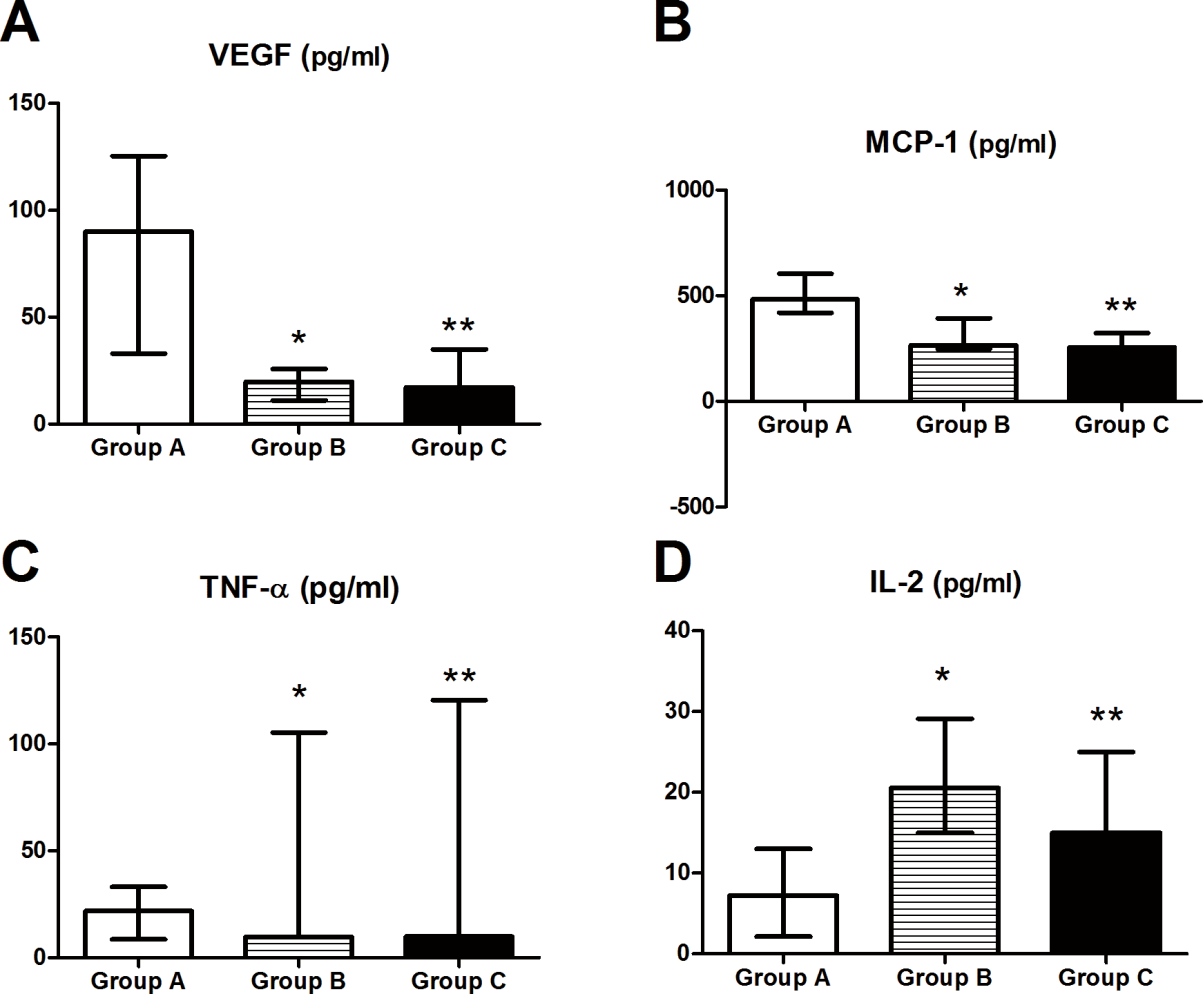
Aqueous humor concentrations of inflammatory cytokines prior to final treatment in the three study groups (mean, median, SD).

Significantly different cytokine levels (pg/ml) are shown among the three study groups. The VEGF, MCP-1, and TNF-α levels were significantly higher in Group A than in Group C (Fig 2A–2C). The VEGF and MCP-1 levels were statistically higher in Group B than in Group C (Fig 2D and 2E). Significantly higher VEGF and MCP-1 levels were observed in Group A compared to Group B (Fig 2F and 2G). *Statistically significant results in two groups (*P* < 0.05). Group A: refractory PCV, Group B: stable PCV, Group C: control.

To investigate whether cytokine levels in the aqueous humor of patients with PCV can act as markers of refractory disease, we compared the six cytokine levels among the groups prior to the final PCV treatment (i.e. the last IVR) (Table 3). VEGF, MCP-1, and TNF-α levels in Group A were significantly higher than Group B and Group C (versus Group B: *P*_VEGF_ < 0.001, *P*_MCP-1_ < 0.001, *P*_TNF-α_ = 0.025; versus Group C: *P*_VEGF_ < 0.001, *P*_MCP-1_ < 0.001, *P*_TNF-α_ = 0.011, respectively). Furthermore, IL-2 levels in Group A were significantly lower than those in the other two groups (*P <* 0.001 for both) (Fig 2). There was no significant difference in cytokine levels between Group B and Group C.

**Table 3:** Levels of inflammatory cytokines prior to final treatment of PCV.

Group	(pg/ml)	IL-2	IL-6	IL-8	MCP-1	VEGF	TNF-α
Control	Mean	22.01	10.02	14.91	276.59	20.48	16.30
	Median	15.03	8.05	13.87	255.50	17.13	9.96
	SD	24.61	8.93	14.61	94.34	18.42	21.77
Pro-treatment							
Group A	Mean	9.53	9.96	16.28	495.16	88.17	29.49
	Median	7.49	6.29	13.30	458.02	82.49	21.19
	SD	9.33	15.00	14.67	223.90	65.80	37.95
Group B	Mean	23.15	11.74	16.38	868.85	23.76	20.06
	Median	20.45	6.09	8.07	264.45	20.32	9.73
	SD	20.48	17.26	26.59	370.54	21.18	27.65
*P* value		<0.001*	0.706	0.083	<0.001*	<0.001*	0.022*

*P* value means Kruskal-Wallis H test among three groups.

*Statistically significant results in the three groups (*P* < 0.05).

Significantly different cytokine levels (pg/ml) are shown among the three study groups. VEGF, MCP-1, and TNF-α levels were significantly higher in Group A than in the other two groups. The IL-2 levels were significantly lower in Group A than in the other two groups. * *P* < 0.05 versus Group A. Group A: refractory PCV, Group B: stable PCV, Group C: control.

## Discussion

PCV is an ocular neovascular disease that is more prevalent among elderly Asians [10, 11]. PCV and wAMD may share some common mechanisms in their pathogenesis. Recent studies have demonstrated that inflammatory cytokines such as IL-6 and IL-8 may play an important role in the pathogenesis of wAMD [12]. However, the linkage between inflammation and PCV remains largely unknown. Clinical studies have shown that intravitreal triamcinolone acetonide may be beneficial in PCV, suggesting inflammation may be involved in the pathogenesis of the disease [13, 14]. Our study showed that pro-inflammatory cytokines such as VEGF, MCP-1 and TNF-α may be associated with PCV occurrence and persistence. We also found that IL-2 may be a protective factor against PCV.

The role of VEGF in ocular neovascularization has been widely studied. Researchers have shown expression of VEGF in choroidal neovascular membranes collected from PCV patients [15]. Elevated VEGF levels in the aqueous humor were also observed in PCV patients [5, 16]. In the present study, we confirmed that patients with PCV had increased VEGF levels in the aqueous humor, which was consistent with earlier studies [5, 15, 16]. The increased expression of VEGF in PCV may contribute to polyp formation and BVN by promoting angiogenesis and to subretinal fluid accumulation by increasing vascular leakage. This may also explain why VEGF levels in refractory PCV patients were higher than stable PCV patients.

An unexpected result was that higher MCP-1 levels were associated with PCV occurrence and persistence. MCP-1 is derived from leukocyte and stroma cells within the hematopoietic microenvironment, which can induce adhesion of macrophages to vascular endothelial cells. Moreover, increased expression of MCP-1 also causes a reduction of vascular smooth muscle and collagen content and mediates hemodynamic changes [17–20]. A number of molecular and immunological studies have shown that MCP-1 is potentially correlated with the pathogenesis, progression, and prognosis of atherosclerosis (AS) [21]. In fact, histopathology of PCV membranes have been shown to demonstrate vascular hyalinization, suggesting the pathogenesis of PCV is similar to that of AS [22, 23]. Our study also showed higher MCP-1 levels in patients with refractory PCV compared to those with stable disease. This suggests increased choroidal hyalinization may play an important role in refractory PCV. Increased MCP-1 levels may therefore contribute to both occurrence and persistence of polyps and BVN.

Our results also showed that increased levels of TNF-α were associated with both stable and refractory PCV. TNF-α is a pro-inflammatory cytokine which can activate cellular activities via intercellular signal transduction (e.g. phosphorylation). This induces a systemic inflammatory reaction, resulting in changes in hemodynamics, injury to the vascular endothelial cells and damage to target organs [24, 25]. Our findings suggest TNF-α may be a crucial factor in PCV, possibly by increasing vascular leakage.

IL-2 can maintain the activities of regulatory T-cells and mediate immune reactions. IL-2 promotes the differentiation of immature T-cells into regulatory T-cells and also promotes the differentiation of T cells into effector T-cells and memory T-cells after stimulation by an antigen. Its expression and secretion is normally tightly regulated via both positive and negative feedback loops in the immune system. In our study, aqueous IL-2 levels were lower in Group A compared to Group B. The IL-2 levels were not correlated with the number of PDT treatment or IVR treatment in either group (data not shown), although the treatment modalities were not well balanced between the two groups. Therefore, we speculate that IL-2 levels may play an immunosuppressive role in refractory PCV, although this speculation is only preliminary and more solid evidences are needed to prove it. However, specific details about immunosuppression in PCV remain unknown. Our study may offer more evidence for exploring the potential mechanisms of immunosuppression in refractory PCV.

There are several limitations to our study. Firstly, the treatment modalities we used in the two PCV groups were not uniform (IVR alone versus IVR combined with PDT). Since combination treatment with PDT and IVR may have different effects on cytokine expression compared to IVR alone, our results may merely reflect the difference in treatment modalities, rather than a difference in disease status. This is the main drawback of our study. However, the proportion of patients treated with IVR monotherapy or IVR combined with PDT was not significantly different between Group A and Group B. The average number of total PDT treatment for each patient was not significantly different between the two groups, either. Moreover, it has been shown that IVR combined with PDT does not cause more aqueous VEGF reduction compared to IVR alone in patients with wAMD[26]. PDT only causes a short-term reduction in VEGF and other inflammatory markers. This may be because the cells producing VEGF and these inflammatory markers begin to recover one week after PDT[27, 28]. The average number of total IVR treatment for each patient was higher in Group A compared to Group B. Despite having received more IVR treatment, patients in Group A still had higher aqueous VEGF levels before the final treatment, suggesting the role of VEGF in the pathogenesis of refractory PCV. Due to the small sample size, it is impossible to compare the cytokine levels after adjusting for treatment modalities in our study. Recruiting PCV patients treated with the same treatment regimen would improve the internal validity of the study. Secondly, our study is a retrospective design, with all the inherent associated biases. Well-designed prospective randomized controlled studies are needed to verify our conclusions.

In summary, our study demonstrated higher levels of certain inflammatory cytokines—such as MCP-1, TNF-α, and VEGF—in patients with PCV compared to the control group. Expression of these cytokines was also higher in refractory PCV patients compared to those with stable disease. These findings suggest that inflammatory cytokines may be involved in the pathogenesis of occurrence and persistence of PCV. Our analysis of only a limited number of inflammatory factors cannot completely demonstrate the mechanisms involved in the pathogenesis of PCV. However, we have demonstrated some key inflammatory pathways that are implicated in PCV, on which further studies can focus to fully understand the immunological and immunosuppresive mechanisms behind the disease.
